# Identification of a BRCA2 mutation in a Turkish family with early‐onset breast cancer

**DOI:** 10.1002/ccr3.1625

**Published:** 2018-07-17

**Authors:** Elifnaz Celik, Kubra Ermis Tekkus, Izzet Mehmet Akcay, Gizem Alkurt Sal, Fikret Ezberci, Gizem Dinler Doganay, Levent Doganay

**Affiliations:** ^1^ Department of Molecular Biology‐Genetics & Biotechnology Istanbul Technical University Istanbul Turkey; ^2^ GLAB (Genomic Laboratory) Istanbul Association of Northern Anatolian Public Hospitals Istanbul Turkey; ^3^ Department of General Surgery Umraniye Teaching and Research Hospital University of Health Sciences Istanbul Turkey; ^4^ Department of Clinical Genetics Umraniye Teaching and Research Hospital University of Health Sciences Istanbul Turkey; ^5^ Department of Gastroenterology and Hepatology Umraniye Teaching and Research Hospital University of Health Sciences Istanbul Turkey

**Keywords:** *ATM*, *BRCA1*/*2*, early‐onset breast cancer, genetics, multi‐gene panel testing, oncology

## Abstract

We used a multi‐gene panel testing to identify the germline variants in a mother‐daughter pair with early‐onset breast cancer, and detected one pathogenic protein‐truncating variant in *BRCA2*. Our results highlight the importance of genetic testing in identifying the pathogenic mutation running in cancer families.

## 1 INTRODUCTION

Breast cancer is the most common and deadly cancer among women.^1^ The vast majority of breast cancers are sporadic, arising from somatic mutations, whereas 10% of all breast cancer cases are hereditary, clustering in families and having an early onset. Deleterious germline mutations in BRCA1 and BRCA2 genes are the most important risk factors for hereditary breast and ovarian cancer, which is inherited in an autosomal dominant manner. Individuals with these germline mutations have a lifetime risk of developing breast cancer of 50‐80%.^2^ *BRCA1* and *BRCA2* are responsible for up to 25% of all familial breast cancers.^3^ Additional cancer predisposition genes (e.g. *ATM, PALB2, TP53, BARD1, CHEK2*) are also implied in hereditary breast cancer.^3^

Most breast cancer predisposition genes have functions in maintaining genome integrity and cell cycle control. ATM, a serine/threonine protein kinase, functions as a transducer of DNA damage signals, and activates downstream proteins, including BRCA1 and BRCA2, by phosphorylation.^4^ BRCA1 is an E3 ubiquitin protein ligase and a transcriptional activator. It plays a central role in coordinating cellular pathways in response to DNA damage. Most notably, BRCA1 stimulates DNA repair mechanisms, and arrests cell cycle progression to ensure that DNA is repaired before division.^5^ BRCA2 is an ssDNA binding protein, and has a vital role in DNA damage response by regulating homologous recombination.

Genetic tests are recommended for individuals suspected to have germline variants. The results of these tests might be important for personalizing the management of the disease and take preventive measures for the subjects and their families. With multi‐gene panel testing, germline variants in the exons of many cancer predisposing genes can be screened in a run in a cost‐effective manner. In this case report, we studied a Turkish mother‐daughter pair with early‐onset breast cancers using a multi‐gene panel and identified two variants of uncertain significance (VUS) in *ATM*,* BRCA1* and a pathogenic variant in *BRCA2*.

## 2 MATERIALS AND METHODS

**Figure 1:**
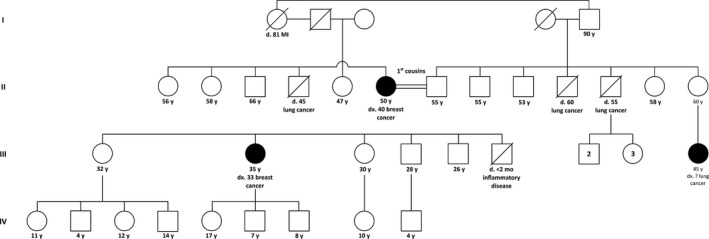
Pedigree of the index patient with early‐onset breast cancer

The index patient (the daughter) was recruited in the Surgical Oncology Department of Umraniye Teaching and Research Hospital (UEAH), Istanbul in 2015. Blood samples from the index and her mother, who was also found to have had early‐onset breast cancer, were collected and subsequent genetic tests were performed in the joint Genomic Laboratory (GLAB) of UEAH and Istanbul Technical University.^6^ Genomic DNAs were isolated using PureLink Genomic DNA Mini Kit (Thermo Fisher Scientific) from blood samples of the index patient and her mother. DNA libraries were prepared using TruSight Cancer Kit (Illumina), and sequenced in MiSeq sequencer (Illumina), using 2 × 150‐bp paired‐end reads. Sequence assembling and variant calling were done using Sophia DDM software (Sophia Genetics). SIFT, PolyPhen‐2, MutationTaster, Provean, and Mutation Assessor tools were also used to predict the pathogenicity of missense variants.^7^, ^8^, ^9^, ^10^ The family history of cancer was interrogated by medical geneticists at UEAH (Figure 1). The study was carried out with the given consent of the patients and the approval of the ethical committee of UEAH (No: 49/24.03.2016).

## 3 RESULTS

The mother‐daughter pair studied in this case report developed breast cancers at young age, suggesting an underlying germline mutation. The daughter was diagnosed with bilateral triple negative invasive ductal carcinoma at the age of 33 in 2015, and had bilateral modified radical mastectomy. Cancer has metastasized to the brain; and the patient is currently receiving chemotherapy. The mother was diagnosed with breast cancer at the age of 40 in 2006, had unilateral mastectomy, and is currently alive. Both patients were referred for genetic testing. Pathological information of the index is described in Table 1.

**Table 1:** Pathological information of index

	Tumor type	Histological grade	Nuclear grade	Tumor localization	ER/PR/cERB2	In situ components	Lenf	T	N	M	V	R	L
Right breast	Invasive breast carcinoma with extensive in situ components	3+2+1:6 II/III (Bloom & Richardson modified)	II/III (Black modified)	Lower outer quadrant	−/−/+	90% high grade w/& w/o necrosis	35/48 carcinoma metastasis, 13/48 reactive hyperplasia	Pt1a	N3a	X	0	0	1
Left breast	Invasive breast carcinoma with micropapillary differentiation	2+2+1:5 I/III (Bloom & Richardson modified)	II/III (Black modified)	6 o'clock position	+/+/+	75% high grade w/& w/o necrosis	1/10 carcinoma metastasis, 9/10 reactive hyperplasia	Pt1b	N1a	X	0	0	1

Three germline variants, common to both patients, were detected using a multi‐gene panel. The variants in *ATM* (NM_000051.3c.8965C>G:p.Gln2989Glu) and *BRCA1* (NM_007294.3c.3424G>C:p.Ala1142Pro) cause nonsynonymous amino acid changes. The clinical significance of these variants is not known. Further analysis is needed to assess their pathogenicity. On the other hand, the variant in *BRCA2* (NM_000059.3c.7655_7658delTTAA:p.Ile2552Thrfs) causes a premature stop codon, leading to the truncation of the C‐terminal 866 amino acids in BRCA2 protein. Truncating variants in BRCA2 are highly pathogenic for breast cancer. Variants in *BRCA1* and *BRCA2* genes were also confirmed by Sanger sequencing.

## 4 DISCUSSION

The germline variant in *ATM* (8965C>G) results in the change of glutamine to glutamic acid at position 2989, which is located in the FATC domain of ATM.^11^ FATC domain is the binding region for Tip60. The interaction between ATM and Tip60, is important for the activation of *ATM*.^12^ In silico prediction tools (SIFT, PolyPhen, MutationTaster, Provean, Mutation assessor) showed conflicting interpretation for this variant.

The variant in *BRCA1* results in the change of alanine to proline at position 1142, which is not located in any known functional domain. Proline leads to rigid turns in protein secondary structures, hence missense variants including proline might affect protein folding and structure. However, in silico prediction tools showed conflicting interpretation for this variant.

TTAA deletion in exon 16 of *BRCA2* (NM_000059.3c.7655_7658delTTAA:p.Ile2552Thrfs) causes a frameshift starting from codon 2552 and leading to a stop codon after adding 95 amino acids. The wild‐type BRCA2 protein is 3418 amino acid long. Thus, this variant is predicted to be highly pathogenic. The resulting truncated protein would be devoid of very important functional domains, including the SEM1‐binding site, DNA‐binding site, nuclear localization signal (NLS), and the CDK phosphorylation site at S3291, which also binds RAD51.^2^, ^13^ SEM1 stabilizes BRCA2 by preventing its degradation. *In vitro* studies showed that loss of SEM1 binding to BRCA2, or depletion of either protein, led to hypersensitivity to DNA damage.^13^ DNA‐binding site is responsible for binding of BRCA2 to single‐stranded DNA and acting as a junction between single‐strand and double‐strand DNA to manage Rad51‐mediated homolog recombination.^14^ Deletion of NLS site causes aberrant localization of BRCA2, preventing its function in maintaining the integrity of DNA and leading to carcinogenesis.^15^ RAD51 directly binds ssDNA and recruitment is provided by BRCA2. RAD51 binding to BRCA2 at the C‐terminus is dependent on the phosphorylation of serine at 3291 by CDK.^2^

Premature termination codons (PTCs) introduced by *BRCA2* mutations also causes degradation of the *BRCA2* mRNA by nonsense‐mediated mRNA decay (NMD), a protective mechanism that prevents the expression of truncated proteins. PTC‐containing BRCA2 transcripts are significantly less prevalent than their counterparts. Therefore, NMD mechanism recognizes PTCs in BRCA2 transcripts and leads to their degradation.^16^ Reduced BRCA2 levels are associated with cancer, as loss of either BRCA allele is frequently observed in breast cancer tumors of *BRCA1* and *BRCA2* mutation carriers.^2^

*BRCA2* variant 7655_7658delTTAA was previously reported in the ClinVar database in Polish, Chinese and New Zealander breast cancer patients.^17^, ^18^ Now, we report that this pathogenic variant is also found in a Turkish breast cancer family. Deleterious BRCA2 variants also predispose to ovarian cancer, and might occur in families with Fanconi anemia. However, we did not find any member of the family with these diseases. Furthermore, no other breast cancer case was found in the maternal side of the family, suggesting that the pathogenic *BRCA2* variant might be a *de novo* germline mutation in the mother of the index. However, we could not receive the consent of the maternal aunts of the index to test this.

Apart from the pathogenic BRCA2 variant, both the index and her mother carry the same two VUS in *ATM* and *BRCA1*. Hence, we cannot infer the clinical significance of these VUS on the basis of the limited data we have. Strikingly, the consanguineous family presented here has an aggregation of lung cancers (Figure 1). Two paternal uncles of the index had lung cancer; and the paternal cousin and the maternal uncle had early‐onset lung cancer. Despite that the uncles of the index were found to be heavy smokers, frequent presentation of lung cancer in the family might also indicate a genetic predisposition. A germline mutation spectrum study in 555 lung adenocarcinoma cases found deleterious variants in *ATM* and *BRCA2* genes, suggesting that inherited risk factors might lead to lung adenocarcinoma.^19^ Three of the lung cancer patients in the family have already died, and the paternal cousin was not reached. Therefore, we were not able to examine the cosegregation pattern of lung cancer with the two VUS in *ATM* and *BRCA1* genes, and the pathogenic variant in *BRCA2* to test the link between these genes and lung cancer.

In conclusion, in this case report we provide another evidence for the pathogenicity of truncating germline *BRCA2* variants in breast cancer.

## CONFLICT OF INTEREST

None declared.

## AUTHOR CONTRIBUTION

EC: and IMA: wrote the manuscript. KET: collected the data for both mother and daughter. GAS: did the experiments. FE: performed the surgery of daughter. GDD: and LD: edited the manuscript.
